# Hidden in plain sight: A decades-long journey of a nasal foreign body discovered incidentally

**DOI:** 10.1016/j.ijscr.2025.111180

**Published:** 2025-03-20

**Authors:** Firas K. Almarri, Khalid S. Abdulsalam, Shahad M. Almohanna, Abdulrahman Awad Alserhani

**Affiliations:** a Department of Otorhinolaryngology – Head and Neck Surgery, Ad Diriyah Hospital, Third Health Cluster, Riyadh, Saudi Arabia; b Department of Otolaryngology – Head and Neck Surgery, King Saud University, Riyadh, Saudi Arabia

**Keywords:** Nasal foreign body, Asymptomatic, Incidental discovery, Patient education, Case report

## Abstract

**Introduction:**

Nasal foreign bodies (NFBs) are commonly encountered in pediatric patients but rarely persist asymptomatically into adolescence. Typically, NFBs present with symptoms such as nasal obstruction, rhinorrhea, or foul odor. However, asymptomatic cases pose diagnostic challenges and are seldom identified without incidental findings.

**Case presentation:**

We present the case of a 17-year-old male with an asymptomatic NFB discovered incidentally during routine dental radiography. The patient denied any history of nasal obstruction, epistaxis, or discomfort. Imaging revealed a radiopaque object in the right nasal cavity, later identified as a metallic snap button embedded in the floor of the nasal cavity. The foreign body had likely been retained for over a decade.

**Clinical discussion:**

The asymptomatic nature of this case challenges the typical presentation of NFBs. The inert material of the foreign body and its location within the nasal cavity likely contributed to the absence of symptoms. This case underscores the importance of considering NFBs in differential diagnoses, even when patients remain asymptomatic. Dentists, in particular, play a critical role in identifying such foreign bodies during routine imaging, as they may otherwise go unnoticed.

**Conclusion:**

This case contributes to the limited literature on long-standing, asymptomatic nasal foreign bodies. It emphasizes the need for healthcare providers, including dentists, to remain vigilant and consider foreign bodies in cases of incidental findings. Appropriate follow-up and education are necessary to prevent potential complications associated with untreated foreign bodies.

## Highlights

• A nasal foreign body (NFB) was incidentally discovered in a 17-year-old male.
• A metallic snap button was identified during routine dental imaging.
• The asymptomatic nature challenges typical NFB presentations.
• Dentists play a key role in detecting NFBs via routine imaging.
• Patient education ensures follow-up, preventing long-term complications.

## 1 Introduction

Nasal foreign bodies (NFBs) are common in pediatric populations, often leading to various complications if left undiagnosed. These objects can be accidentally inserted into the nasal cavity, particularly in young children or, in rare cases, even adults [1]. While the acute presentation of NFBs often includes symptoms such as nasal obstruction, rhinorrhea, and foul odor, cases where the foreign body remains asymptomatic for extended periods are rare and pose significant diagnostic challenges [2,3].

In particular, chronic NFBs can lead to complications such as rhinolith formation, erosion of nasal structures, and secondary infections [3]. The discovery of such objects is often incidental during routine medical or dental examinations, as seen in this case report [4,5]. The rarity of such cases emphasizes the importance of maintaining a high index of suspicion, even in asymptomatic patients, particularly when unusual findings are detected on radiographic imaging [6].

Herein, we report a unique presentation of a NFB retained for over a decade, discovered incidentally during a routine dental examination. Despite its long-standing presence, the patient remained asymptomatic, highlighting the potential for such objects to go undetected for years and the importance of thorough diagnostic evaluation when incidental findings occur. This case report has been reported in line with the SCARE 2023 criteria [7].

## 2 Case presentation

**Fig. 1:**
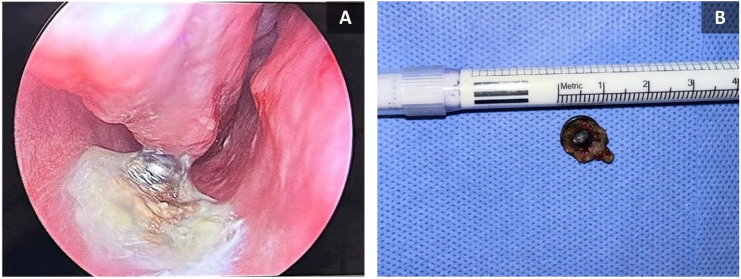
(A) An endoscopic image of the right nasal cavity showing a lodged foreign body underneath the inferior turbinate with an edematous and erythematous mucosa, (B) The retrieved metallic snap button with signs of substantial corrosion and biological deposits.

**Fig. 2:**
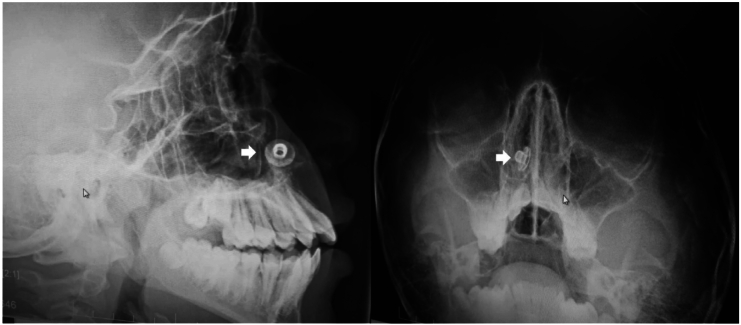
Lateral and frontal X-rays views showing a circular, radiopaque object situated along the nasal floor *(arrows)*.

**Fig. 3:**
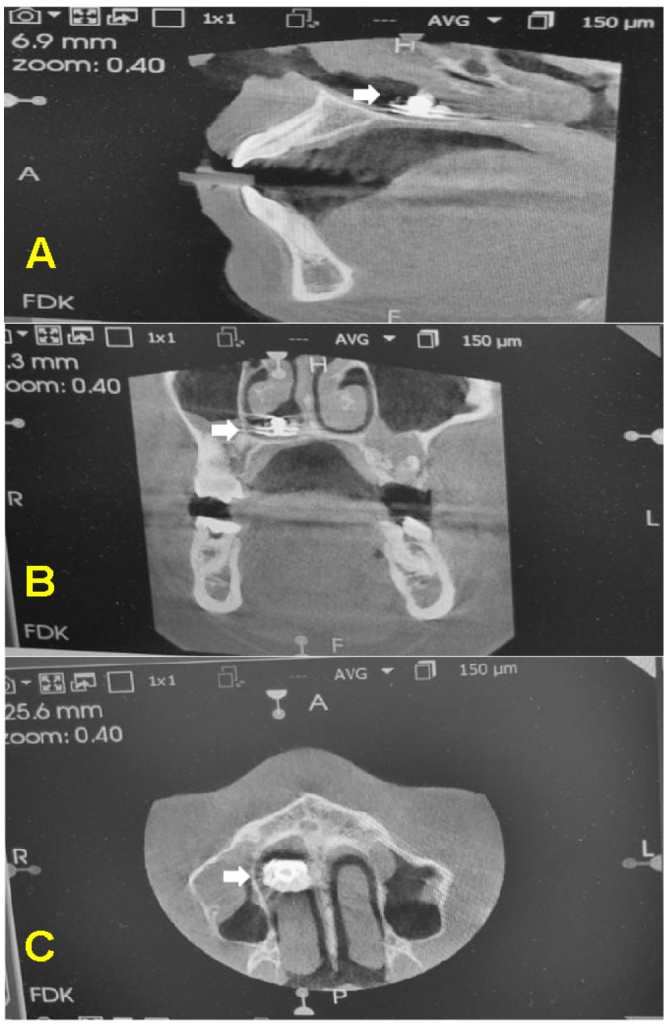
Cone beam computed tomography (CBCT) without contrast (**A;** Sagittal, **B;** coronal and **C;** Axial views) showing a well-defined, circular, hyperdense object located posteriorly *(arrows)*.

A 17-year-old male with no significant past medical or family history was referred to our clinic from the dental department following an incidental finding of a NFB during preoperative orthodontic planning, including dental x-rays and cone beam computed tomography (CBCT) without contrast. The patient was entirely asymptomatic and denied any history of nasal obstruction, rhinorrhea, epistaxis, foul odor, hyposmia, halitosis, facial pain, discomfort, or sleep disturbances. The patient's parents recalled an event when their son was seven, where he inserted an object into his nose. They sought medical advice, where no imaging was performed and an anterior rhinoscopy was utilized for diagnoses but due to the child's non-cooperation during the examination, the physician recommended the removal of the foreign body under sedation. However, the family did not follow up, and since the child remained asymptomatic, they assumed the foreign body had fallen out on its own. On endoscopic examination of the right nasal cavity, a deviated nasal septum with inferior turbinate hypertrophy was noted. The mucosa appeared erythematous and slightly edematous. A foreign body was visualized, lodged, and adhered to the floor of the nasal cavity beneath the inferior turbinate. The object was partially covered with mucus and possibly some crusted material and had a shiny appearance, indicating a metallic nature (Fig. 1, A). Radiographic evaluation, including lateral and frontal X-rays, revealed a circular radiopaque object consistent with a metallic snap button located along the floor of the nasal cavity (Fig. 2). The surrounding bony structures appeared normal. A CBCT confirmed the presence of the foreign body with associated mild inflammation, but no significant bony damage or sinus involvement was observed (Fig. 3). With informed consent from the patient's parent, the foreign body was removed under local anesthesia in a semi-sitting position to reduce the risk of dislodgment to the airway. After decongesting the nasal cavity with Xylometazoline 0.1 % and administering Lidocaine spray (10 mg/spray) in the right nostril, a hook was utilized to disengage the foreign body, which was then retrieved using bayonet forceps. The procedure was uncomplicated, with minimal bleeding, easily controlled with saline irrigation. Post-removal examination showed no significant tissue damage. The retrieved object, a metallic snap button measuring 1 cm in diameter, exhibited signs of long-term exposure, including substantial corrosion and biological deposits (Fig. 1, B). The patient was discharged in stable condition and prescribed nasal rinses with a sodium chloride irrigation solution (0.9 %). The patient was doing well at his two-week follow-up with an unremarkable examination.

## 3 Discussion

This case of a 17-year-old male with a long-retained NFB discovered incidentally during a routine dental examination highlights several important aspects of otolaryngological practice. The patient had unknowingly harbored a metallic foreign object in his nasal cavity for over a decade without experiencing any symptoms, an occurrence that, while rare, is not unprecedented in the literature.

The asymptomatic nature of this case is particularly noteworthy. As documented in previous studies, most NFBs present with symptoms such as nasal obstruction, purulent discharge, or foul odor, often leading to early detection [3,6,8]. However, cases where foreign bodies remain undetected for many years, as seen in this patient, challenge the typical clinical expectations. [9] Hulse et al. reported a similar case in which a NFB remained undetected for years, eventually causing significant anatomical changes [2]. These findings underscore the potential for chronic NFBs to remain clinically silent, particularly when the object is composed of inert material, as was likely the case here. Moreover, the mechanism by which these foreign bodies can remain asymptomatic is often attributed to their inert nature and the specific location within the nasal cavity that minimizes irritation or obstruction [6]. The absence of symptoms in this patient may also be due to the lack of secondary infections or significant inflammatory responses commonly associated with long-standing foreign bodies [10]. Unlike organic materials, metallic objects are less prone to induce mineral deposition and the formation of rhinoliths. The smooth surface of the snap button likely limited the accumulation of debris, which is typically required for rhinolith formation. Additionally, its location along the floor of the nasal cavity, without obstructing airflow or irritating mucosal structures, contributed to its silent presence. Had the foreign body remained undetected, potential long-term complications could have included chronic inflammation, secondary bacterial infections, septal perforation, or even migration into the airway. The absence of these complications in this case highlights the variability in foreign body responses.

In this case, the incidental discovery of the foreign body emphasizes the importance of thorough evaluation and a multidisciplinary approach when unusual findings are encountered during routine imaging. Additionally, it underlines the important role dentists can have in diagnosing foreign bodies in children as they frequently perform imaging for orthodontic assessment making them key contributors to early detection of asymptomatic NFBs [11]. This case also highlights the role of healthcare providers in educating patients and caregivers about the potential risks of untreated foreign bodies. The patient's failure to follow up after the foreign body was first identified reflects a broader issue in clinical practice where patients or caregivers may dismiss asymptomatic cases. As the literature shows, timely intervention prevents complications such as rhinolith formation, mucosal damage, or sinus involvement [2,6].

Given the rarity of such cases, further research is needed to understand better the factors contributing to the asymptomatic retention of NFB over extended periods. Developing guidelines for managing incidentally discovered foreign bodies, particularly those identified through dental or other routine radiographic examinations, could improve patient outcomes by ensuring prompt and appropriate care. Furthermore, this case underscores the importance of maintaining a high index of suspicion for NFB, even in asymptomatic patients, and highlights the need for careful follow-up when such foreign bodies are suspected. This case adds to the literature by demonstrating that some foreign bodies may remain asymptomatic for over a decade without causing significant structural changes. Such findings challenge existing expectations regarding long-term foreign body retention and warrant further investigation.

## 4 Conclusion

This case highlights the unusual scenario of a long-retained NFB that remained asymptomatic for over a decade. The incidental discovery of the foreign body during a routine dental examination underscores the importance of maintaining a high level of clinical suspicion, even in the absence of symptoms. The rarity of such cases emphasizes the need for vigilance and thorough evaluation when foreign bodies are suspected, particularly in pediatric patients. It also underscores the importance of patient and caregiver education regarding the potential risks of untreated foreign bodies and the necessity of follow-up care.

## Consent

Written informed consent was obtained from the patient's parents/legal guardian for publication of this case report and accompanying images. A copy of the written consent is available for review by the Editor-in-Chief of this journal on request.

## Ethical approval

Not required.

## Guarantor

The guarantor is also the corresponding author.

## Research registration number

Not applicable.

## Provenance and peer review

Not commissioned, externally peer-reviewed.

## Author contributions

FKA: Conceived the study design; FKA and KSA: performed the literature review; FKA, SMA, and AAA: prepared the components of the case presentation; FKA: drafted the manuscript, which AAA then reviewed. All authors read and approved the final version of the manuscript.

## Sources of funding

This study did not receive any sources of funding.

## Declaration of competing interest

The authors state that they have no conflicts of interest regarding the publication of this report.

## Data Availability

The data used to support the findings of this study are included within the article.
